# Can an android’s posture and movement discriminate against the ambiguous emotion perceived from its facial expressions?

**DOI:** 10.1371/journal.pone.0254905

**Published:** 2021-08-10

**Authors:** Satoshi Yagi, Yoshihiro Nakata, Yutaka Nakamura, Hiroshi Ishiguro

**Affiliations:** 1 Department of Engineering Science, Osaka University, Toyonaka, Osaka, Japan; 2 JST ERATO, Chiyoda-ku, Tokyo, Japan; University Hospitals Tubingen: Universitatsklinikum Tubingen, GERMANY

## Abstract

Expressing emotions through various modalities is a crucial function not only for humans but also for robots. The mapping method from facial expressions to the basic emotions is widely used in research on robot emotional expressions. This method claims that there are specific facial muscle activation patterns for each emotional expression and people can perceive these emotions by reading these patterns. However, recent research on human behavior reveals that some emotional expressions, such as the emotion “intense”, are difficult to judge as positive or negative by just looking at the facial expression alone. Nevertheless, it has not been investigated whether robots can also express ambiguous facial expressions with no clear valence and whether the addition of body expressions can make the facial valence clearer to humans. This paper shows that an ambiguous facial expression of an android can be perceived more clearly by viewers when body postures and movements are added. We conducted three experiments and online surveys among North American residents with 94, 114 and 114 participants, respectively. In Experiment 1, by calculating the entropy, we found that the facial expression “intense” was difficult to judge as positive or negative when they were only shown the facial expression. In Experiments 2 and 3, by analyzing ANOVA, we confirmed that participants were better at judging the facial valence when they were shown the whole body of the android, even though the facial expression was the same as in Experiment 1. These results suggest that facial and body expressions by robots should be designed jointly to achieve better communication with humans. In order to achieve smoother cooperative human-robot interaction, such as education by robots, emotion expressions conveyed through a combination of both the face and the body of the robot is necessary to convey the robot’s intentions or desires to humans.

## Introduction

A number of studies have pointed out that nonverbal communication from robots to humans is as important for robots to communicate smoothly with humans in society as it is in human-to-human communication [1–4]. Facial expressions and gestures are representative of the nonverbal communication that people often use to convey their emotions [5, 6]. Therefore, it has been actively studied how robots’ facial expressions and gestures can convey emotions to achieve meaningful communication with humans [7–9]. For example, humanoids’ human-like upper body gestures (nonverbal communication) leads people to perceive a higher animacy of the robot, and the gestures also affect the emotional state and self-disclosure, compared to robot-specific nonverbal behavior (such as LED-eyes color changes) [10]. Also, humanoids’ facial expressions lead to an increase in people’s desire to interact again with the robot [11]. While there are many findings showing that nonverbal expressions by robots play an important role in human-robot communication, it is also reported that slight differences in expression can lead to the conveyance of significant misinformation [12]. Thus, there is still room for further investigation into the implementation of robotic facial expression, and the human perception of such expressions.

It is widely recognized that facial expressions are important for judging human emotions objectively and clearly. In the 19th century, Darwin and Prodger (1998) already described how facial expressions are associated with certain emotions [13]. Subsequently, Ekman et al. (1987) suggested that facial expressions had universality, and classified six types of basic emotions by different facial expressions [14]. Russell (1980) explained the correspondence of facial expressions and emotions by using two dimensional affective scales: valence (positive to negative) and arousal (high to low) [15]. All of this research focused on mapping facial expressions to basic emotions, assuming that there are specific facial muscle activation patterns for each emotional expression, and that other people can perceive these emotions by reading these patterns. This method of mapping facial expressions to certain emotions has also been used in research that enables robots to communicate their emotions to humans. Breazeal (2003) pioneered research on machines imitating emotional expressions, arguing for the importance of facial expressions and eye gaze for a social robot [16]. Later humanoid robots, such as Kobian, Flobi, Bert2, and iCub, would also use this mapping method, with all four having human-like facial features that allowed for basic emotional expressions [17–20]. The android robots developed in our research group, such as Geminoid, took this one step further, with a realistic appearance created by using silicon-made skin and an original actuation mechanism for the face [21]. This allowed the android to express emotions through facial expressions to an almost human degree [22].

Recent research has found some cases in which emotions may not be clearly distinguishable from facial expressions [23]. Meeren, Heijinsbergen, and Gelder (2005) reported that emotional perception is hindered when facial expression differs from body expression (for example, an angry face, but a frightened body posture or vice versa) [24]. Van den Stock, Righart, and Gelder (2007) also reported that the perception of emotions from facial expressions is strongly influenced by body posture [25]. Aviezer, Trope, and Todorov (2012) found that when participants were shown peak expressive reactions to winning and losing points in professional high-stakes tennis matches, it was difficult for participants to correctly judge whether the emotion was positive or negative (i.e. a win or a loss) through intense facial expressions alone, however, they could perceive emotions when these were seen with the body [26]. Aviezer et al. stated that although the faces were inherently ambiguous, viewers erroneously reported perceiving valence in the face and this process seemed to be automatic as participants had little awareness of the actual facial ambiguity and the original diagnostic source of the valence. In addition, later research also showed that intense emotions cannot be judged solely by facial expressions [27].

It was found that humans recognize facial emotions by unconsciously considering factors other than facial expression. In previous research on robots that have emotional expression functions, facial expressions have been used to convey emotions on the assumption that facial expressions have a one-to-one correspondence with certain emotions. Thus, it still has not been investigated whether robots can express the ambiguous facial expressions with no clear valence which humans sometimes show, and what these look like. Furthermore, it has also not been investigated whether the robot’s emotional valence can be clearly determined from such expressions by adding body expressions.

What we validate in this paper are the following: 1) what kind of facial expression of an android is indistinguishable (ambiguous) in the scale of positive to negative emotions?, and 2) with an indistinguishable facial expression, do postures and movements solve the facial ambiguity? We believe that the investigation of ambiguity expressed by the facial expressions of an android and the clear emotional expressions (positive and negative) achieved by adding body modality can help to realize smooth communication between social robots and humans.

Here we present facial expressions, postures and movements of an android used for experiments. As each component of the android’s body can be controlled individually, we conducted three step-by-step experiments and online surveys. In Experiment 1, we validated facial expressions which cannot be clearly discriminated to be either positive or negative, and in Experiment 2 and 3, we validated the possibility of clearly perceiving ambiguous facial expressions of the android by adding postures or movements. In order to investigate only the perception of the peak moment of the android’s emotional expressions, we showed posture photos to participants in Experiment 2. Then, in order to investigate the perception of practical emotional expressions, we showed movement movies to participants in Experiment 3. To assess a perceived emotion, we used the emotional state which consists of two dimensions, Valence (Positive—Negative) and Intensity (High—Low), which is often used in psychological research [28].

## Materials and methods

We used the child-like mobile android that we developed called *ibuki* [29], who is 120 cm tall and comprised of two parts—a mobility unit (lower body, which contains a linear joint that generates a vertical motion of the upper body) [30] and an upper body, both designed based on the dimensions of a 10-year-old Japanese boy. For a human-like appearance, the face and hands are covered with silicone skin. An electric motor drives each of the 47 joints, and mobile batteries are used as the power supply.

The protocol was approved by the ethics committee for research involving human subjects at the Graduate School of Engineering Science, Osaka University (#R1–6). We recruited separate samples of participants using Amazon Mechanical Turk. The number of participants in each assessment is as follows: 94 people in Experiment 1 (41 females and 53 males, Mean age (M) = 35.5 years old, Standard Deviation (SD) = 10.72), 114 people in Experiment 2 (44 females and 70 males, M = 34.9, SD = 9.93), and 114 people in Experiment 3 (53 females and 61 males, M = 35.4, SD = 9.92).

In Experiment 1, participants were asked to answer the following two questions, corresponding to the emotional state consisting of two dimensions: valence and intensity, after viewing *ibuki*’s facial expressions:

1. To rate the robot’s facial emotion (negative to positive valence) from a scale of -4 to 4.
2. To rate the intensity of the robot’s facial expression from a scale of 1 to 9.

In Experiment 2 and 3, participants were asked to watch a photo or movie that showed the moment *ibuki* reacted to the result of a game and instructed to guess whether *ibuki* had won or lost the game based on its facial expression. In addition to the questions from Experiment 1, two more questions were added (interpretation of the game result and human-likeness):

1. To guess whether they think the robot has won or lost the game.
2. To rate the robot’s facial emotion (negative to positive valence) from a scale of -4 to 4.
3. To rate the intensity of the robot’s facial expression from a scale of 1 to 9.
4. To rate the human-likeness of the robot’s facial expression from a scale of 1 to 9.

The question about human-likeness was added to confirm that the human likeness of *ibuki* did not affect the assessment of the valence, since the postures and movements were manually created by the authors.

### Procedure

**Fig 1:**
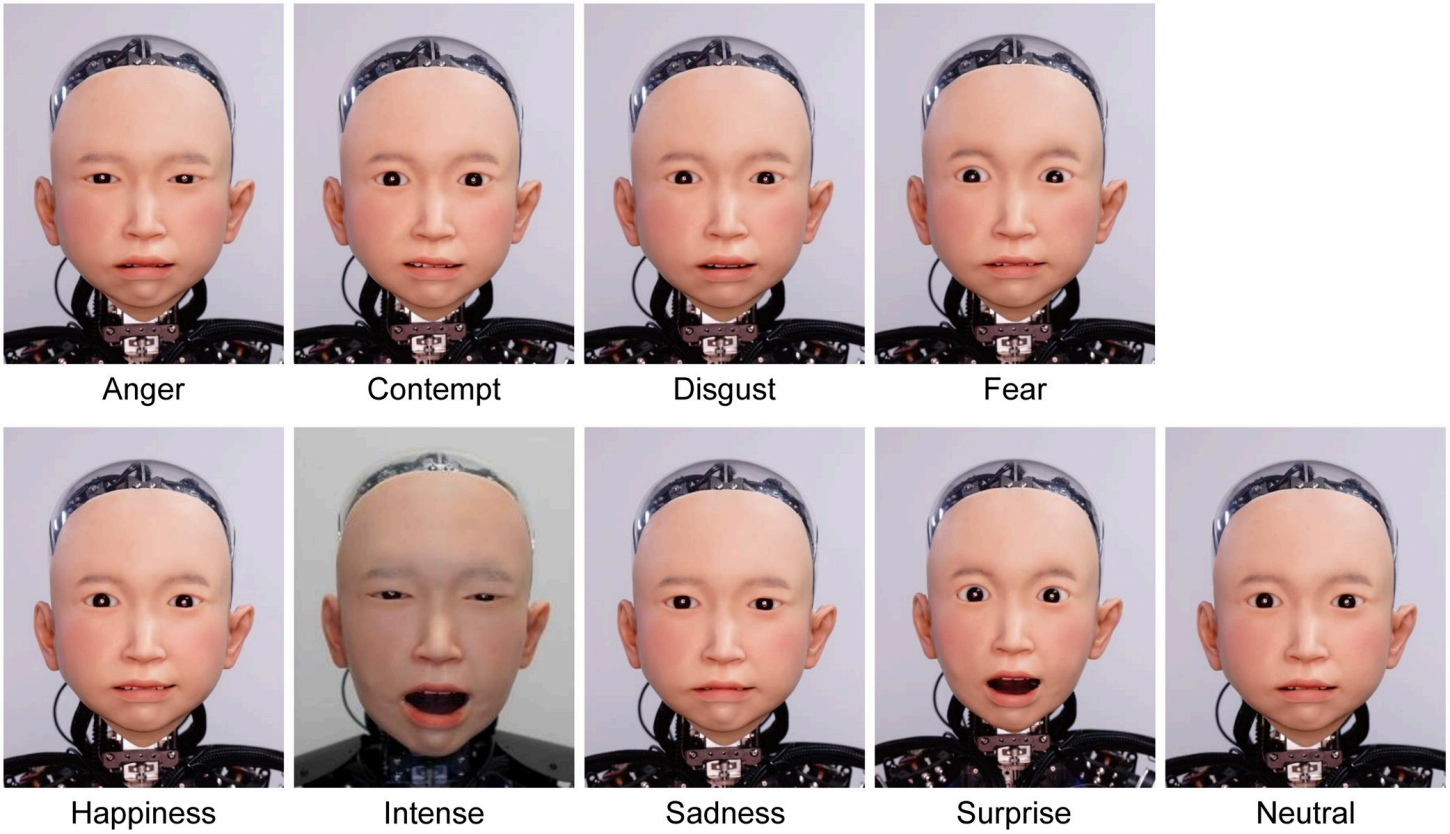
Nine facial expressions of *ibuki*.

In Experiment 1, we investigated whether there are ambiguous facial expressions which cannot be determined by the facial expression alone. For facial expressions, we created nine facial expressions, namely: anger, disgust, fear, happiness, sadness, surprise, contempt, neutral, which are representative emotions in facial expression studies, and intense. For facial expressions except intense, we operated actuators with reference to the Emotional Facial Action Coding System, which explains the characteristics of actual human facial movements. For intense, we created the intense facial expression as an expression of high muscle activity that expresses the excitement immediately after a game result was decided. It was created to look like an emotion in which the eyes are closed strongly and the mouth expresses shouting (See Fig 1) based on the paper of Aviezer et al. [26].

From those eight expressions except neutral, we selected one facial expression with the highest entropy of the valence in order to use it in Experiment 2 and 3. Since entropy is a physical property that represents a state of disorder of the system, for this study, we defined the indistinguishable (ambiguous) facial expression as having the highest entropy of the facial valence distributions. The entropy *S* of each facial expression is calculated by the following equation:

$$\begin{array}{c} S=-\sum_{x_{i}=-4}^{4}P\left(x_{i}\right)\log_{2}P\left(x_{i}\right) \end{array}$$ (1)

where *x*_*i*_ = {−4, −3, ⋯, 3, 4} is the possible facial valence, *P*(*x*_*i*_) is the probability which the facial valence is rated as *x*_*i*_. The calculation method is further detailed in S1 Appendix. In Experiment 1, one photo of the eight emotions (350 x 450 pixels size) was displayed beside the neutral face on each page in a random order. After each viewing, participants were asked to answer the facial valence and intensity by answering the two questions mentioned in the Materials and methods section, taking into account that the valence and intensity of the neutral face was 0 and 1, respectively.

**Fig 2:**
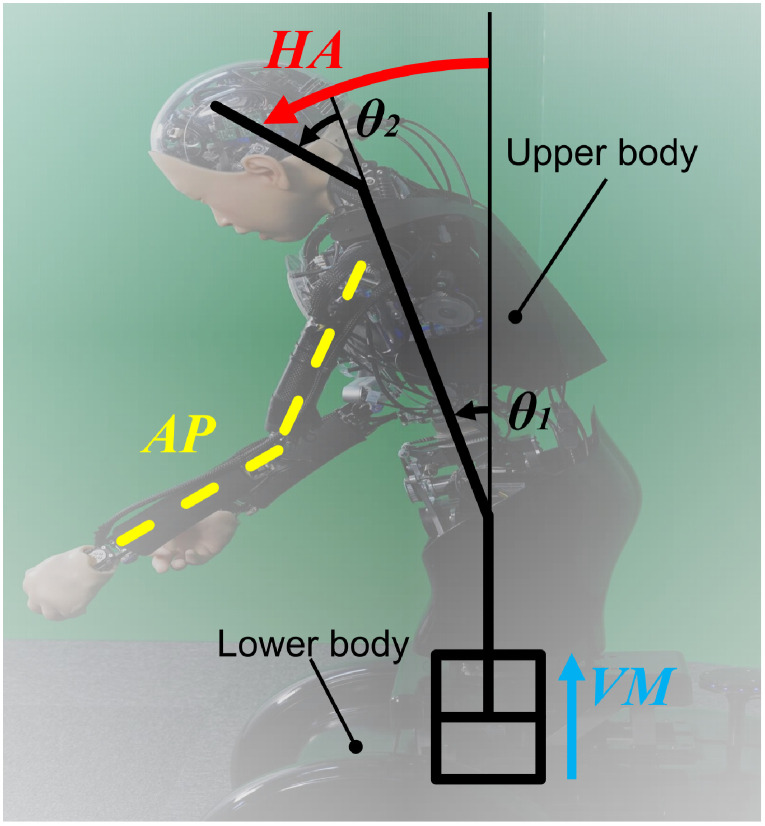
The body expression with the ambiguous facial expression and the configuration of HA, AP, and VM.

Together with the highest entropy facial expression in Experiment 1, we took photos of 15 types of postures by *ibuki* for Experiment 2. In addition, we took movies of 30 types of movements performed by *ibuki*. Firstly, we constructed three arm poses (AP; labeled as A, B, and C) and five head angles (HA; labeled as -43, -17, 0, 17, and 43, which are head inclination degrees at the sagittal plane). HA consist of the neck joint angle *θ*_1_ and waist joint angle *θ*_2_ as shown in Fig 2. Table 1 shows the angles of the head, neck, and waist.

**Table 1:** The relationship among head angle, the neck, and waist joint.

Head angle *HA*	-43	-17	0	17	43
Neck angle *θ*_1_	-30	-30	0	30	30
Waist angle *θ*_2_	-13	13	0	-13	13

All angles are shown in degrees.

**Fig 3:**
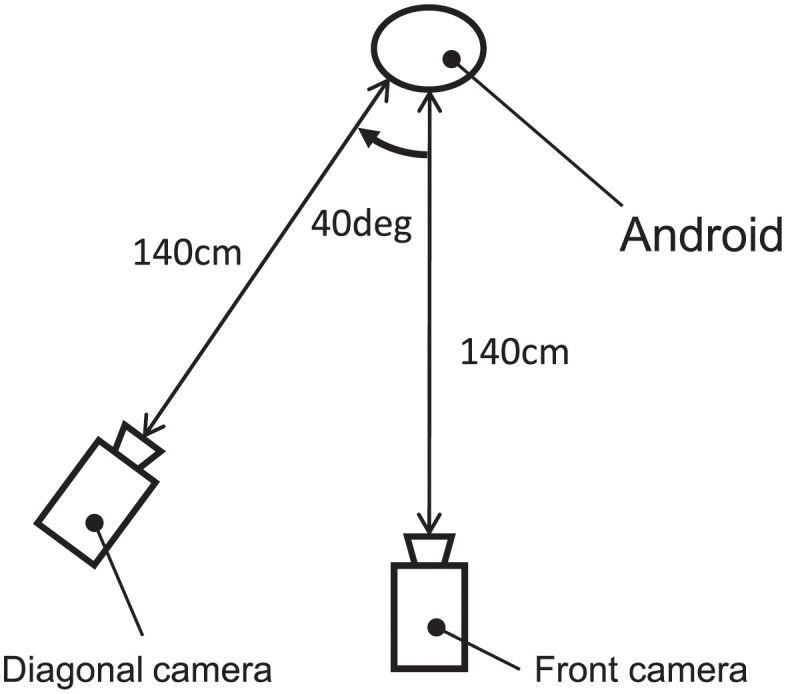
An overview of shooting environment.

**Fig 4:**
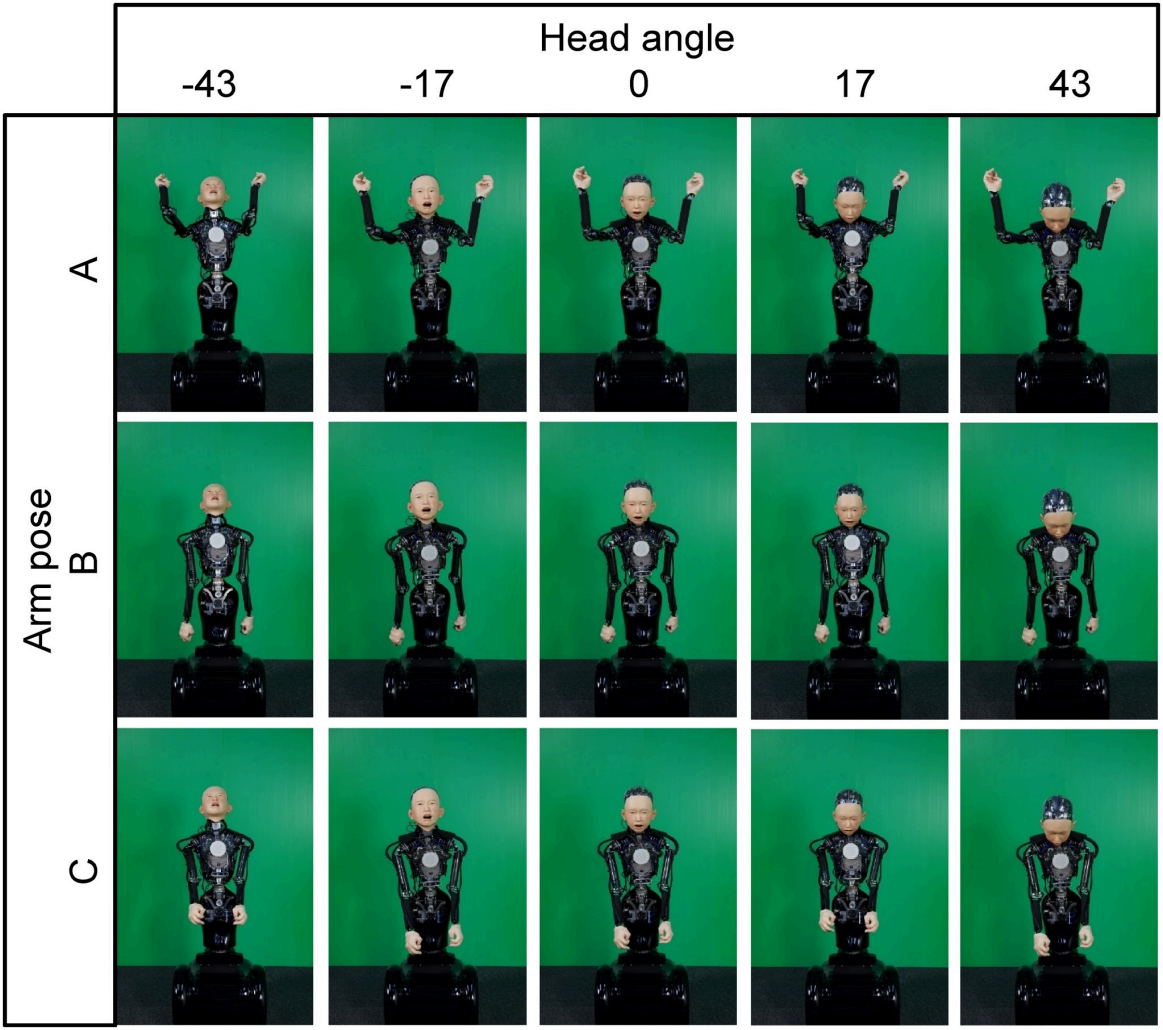
15 postures from the front view.

Secondly, we created 30 movements by combining the previous 15 postures and two types of vertical motions (VM) of the upper body: one was a +40 mm upward motion, the other was a -40 mm downward motion (VM; labeled as 40 and -40). Then, we placed two cameras—in front of and 40 degrees diagonally to *ibuki*—to take photos and movies of *ibuki*’s emotional expressions. Both cameras were installed at a height of 100 cm—assuming the position of an adult on the knees looking at a child 140 cm away (Fig 3). Behind *ibuki* was a neutral green screen. For Experiment 2, we took two photos of each of *ibuki*’s postures from the front and diagonal angles, resulting in 30 photos. Fig 4 shows 15 postures from the front view. For Experiment 3, we took two movies of each movement from the front and diagonal angles, resulting in 60 movies. Each movie was edited to a length of three seconds. The first 0.5 seconds of the movie showed *ibuki* in the neutral posture before moving towards the target posture. Again, the same fixed facial expression was used for both Experiment 2 and 3.

In Experiment 2 and 3, we investigated whether the indistinguishable facial expression can be distinguished by adding body postures and movements. Participants were asked to look at the photos or movies. To eliminate the influence on the assessment of valence due to viewpoint changes, one participant group (57 people) only saw the front angle photos, and the other participant group (57 people) looked at photos taken from the diagonal angle. In order for participants to understand the contents of the questions, we used a context that evaluate the reaction of the child android at the moment when the win or loss of a game was decided. At the beginning of each assessment, the neutral upright posture of *ibuki* was shown as a reference. For both experiments, a photo or movie was displayed on each page in random order one by one. After each viewing, participants were asked to answer the aforementioned four questions (interpretation of the game result, facial valence, intensity and human-likeness).

For the processing of the data of each experiment, only participants who answered the manipulation check questions correctly were counted as valid answers (this question asked participants to answer with a certain score as was instructed in the movie to check if the participants properly watched the movies). In addition, in Experiment 2, the neutral posture with the neutral face that was shown at first as a reference was shown twice during the task, and participants who rated the valence of the neutral posture as -4 or 4 had their answers excluded (resulting in 10 exclusions). In Experiment 3, the movie of condition HA/AP/VM: -17/A/40 was shown three times during the task, and participants who provided answers with a more than five points difference were excluded (resulting in 23 exclusions).

### Analysis

In Experiment 1, distributions of both valence and intensity were analyzed to check the characteristics of *ibuki*’s facial expressions. Then we calculated the entropy for the facial valences of each expression to verify the indistinguishable facial expressions, which have a high entropy.

In Experiment 2, the mean facial valences and entropy were analyzed to check the influence of android body expressions. Two-way repeated measures analyses of variance (ANOVA) were conducted with HA and AP as two within-subjects factors. The significance level was set at 0.05. Partial eta-squared (*η*_*p*_^2^) was reported to demonstrate the effect size in ANOVA. Then Tukey’s HSD test (HSD test) was performed for multiple comparisons to verify our hypothesis that adding postures or movements contributes to the clear perception of the ambiguous facial expression. In addition, the percentage that assessed as *won* at each posture was also calculated to verify the distinguishable face due to body expressions.

As an android can control each component of the body individually—e.g. facial expression, head angles and arm poses—instead of in conjunction with other joints and other parts of the body like humans, the postures and movements of *ibuki* cannot be guaranteed to be intense and human-like. Therefore, we calculated correlations between facial valence with intensity and human-likeness to confirm whether the effect of intensity and human-likeness on the assessment of facial valence was small or not.

In Experiment 3, we analyzed the data following the same analysis procedure used in Experiment 2.

## Results

### Experiment 1

**Fig 5:**
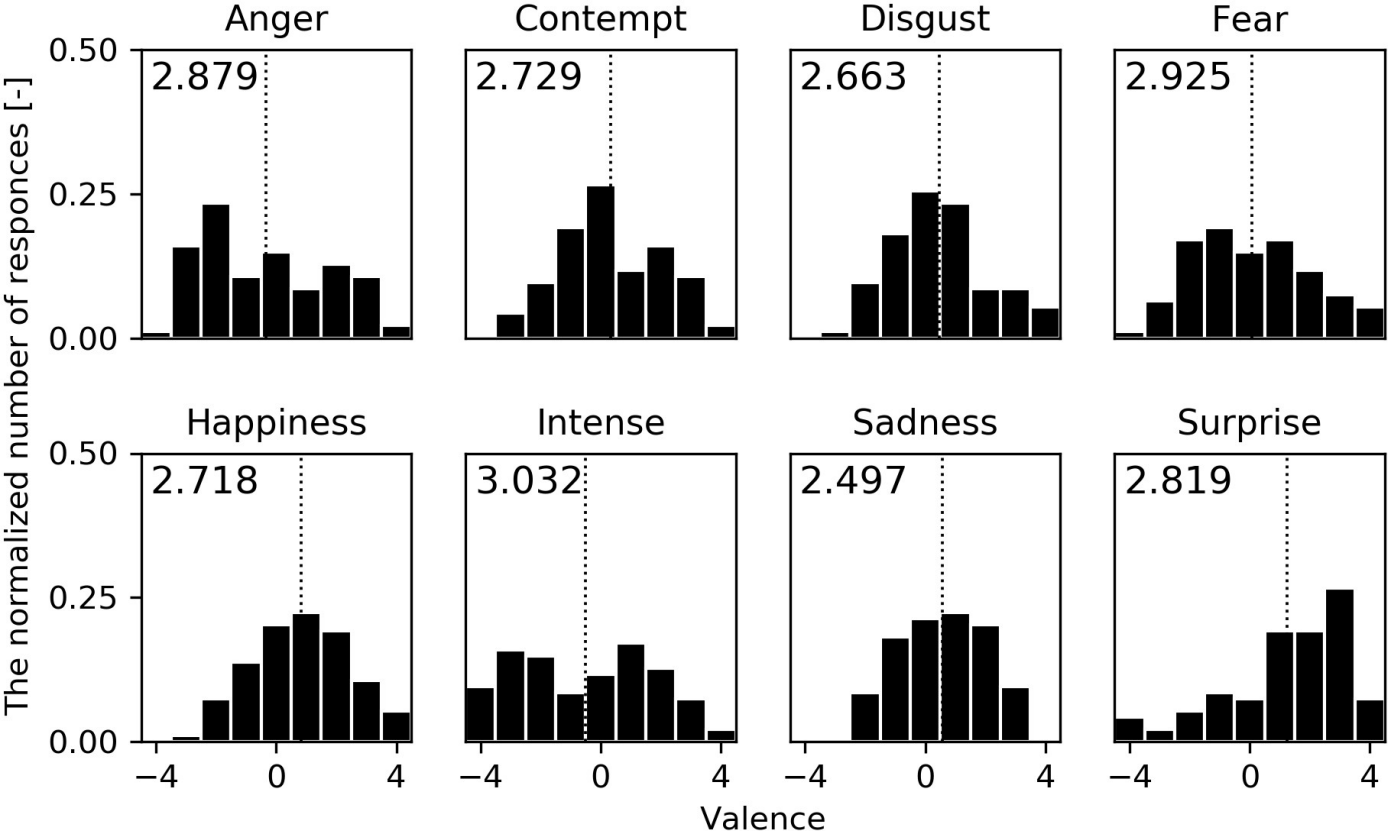
Distributions of the assessed facial valence in Experiment 1. The horizontal axis shows the assessed facial valence (-4 to 4) and the vertical axis shows the normalized number of responses. The dotted line shows the mean facial valence. The entropy is shown in the upper left of each histogram.

Fig 5 shows the distribution of assessed facial valence for eight emotions in Experiment 1. The horizontal axis shows the assessed facial valence (-4 to 4) and the vertical axis shows the normalized number of responses. The dotted line of the histogram shows the mean facial valence. In order to select the most ambiguous face, we calculated the entropy of each distribution, which is shown in the upper left of the histogram. The highest was intense (3.032). With this result, we decided to use intense as the ambiguous facial expression for the next experiments.

**Fig 6:**
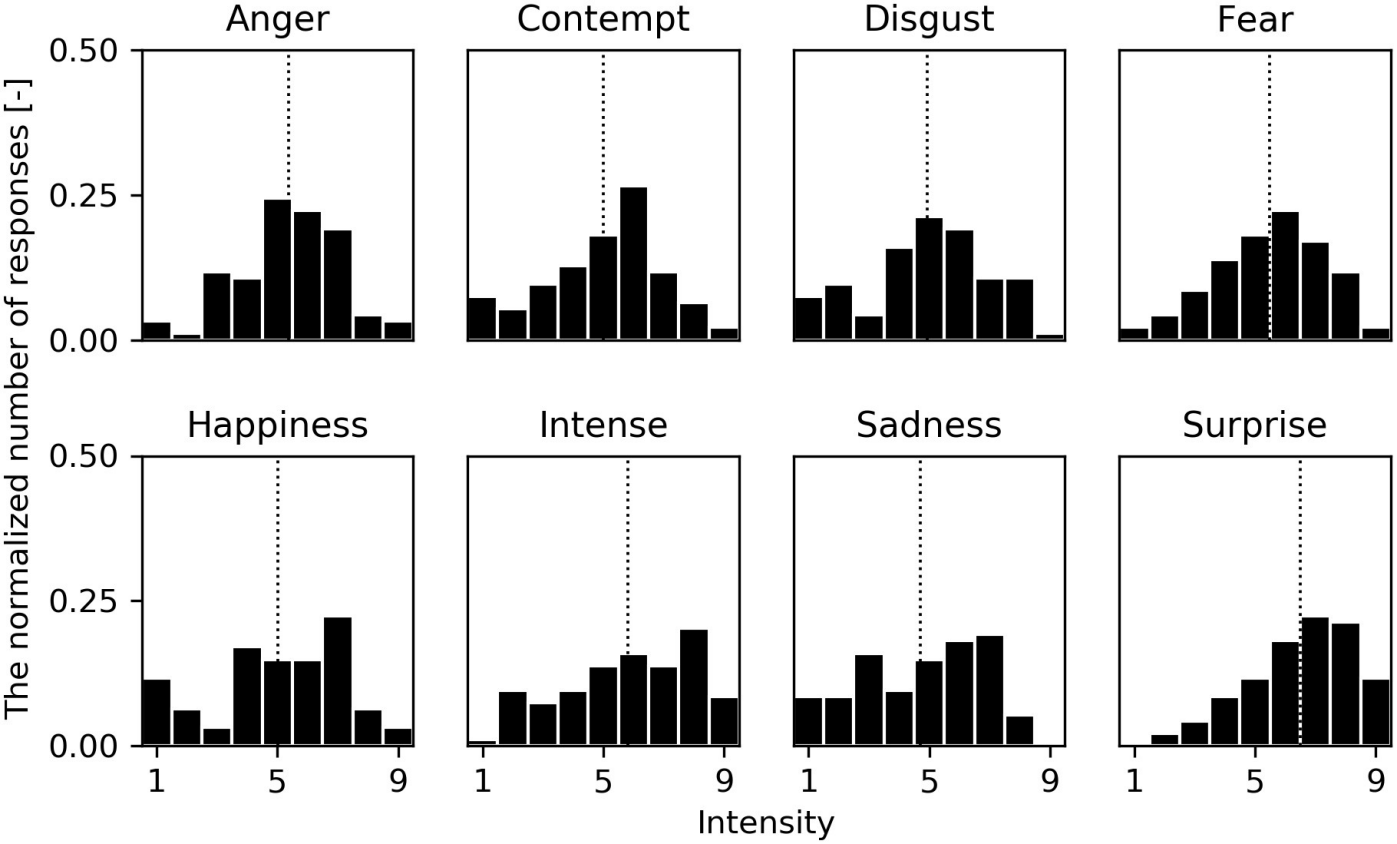
Distributions of the assessed facial intensity in Experiment 1. The horizontal axis shows the assessed facial intensity (1 to 9) and the vertical axis shows the normalized number of responses. The dotted line shows the mean facial intensity.

Fig 6 shows the distribution of assessed facial intensity for the eight emotions in Experiment 1. As in the previous valence result (Fig 5), the horizontal axis shows the assessed facial intensity (1 to 9) and the vertical axis shows the normalized number of responses. The dotted line in the histogram shows the mean facial intensity.

### Experiment 2

**Fig 7:**
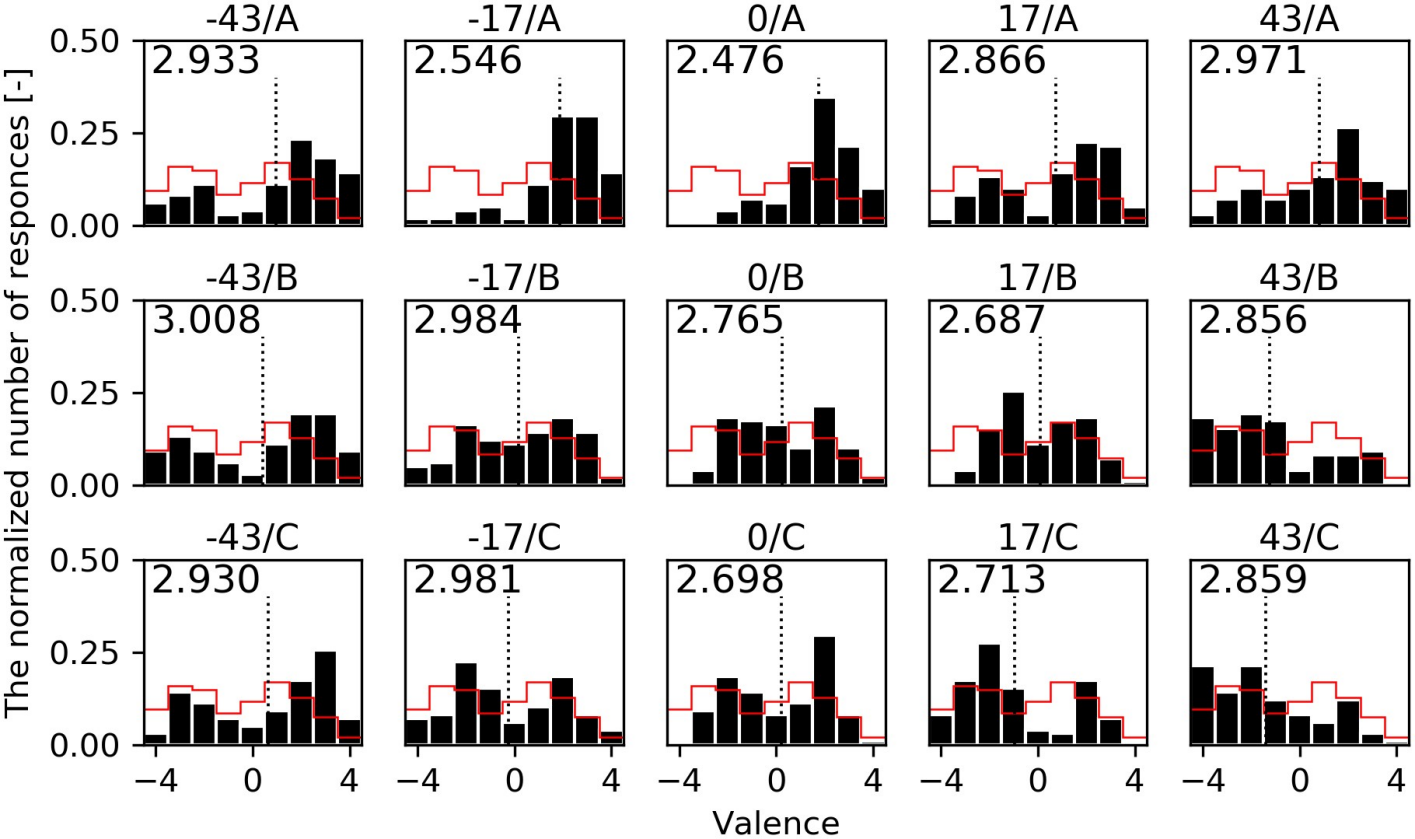
Distributions of the assessed facial valence in Experiment 2. The horizontal axis shows the assessed facial valence (-4 to 4) and the vertical axis shows the normalized number of responses. The dotted line shows the mean facial valence. The red line on the histogram shows the result of the intense facial expressions in Experiment 1 as a reference. The entropy is shown in the upper left of each histogram.

In Experiment 2, *ibuki*’s posture photos were shown to participants in order to investigate only the perception of the peak moment of emotional expressions. We validated that postures contribute to the perception of the indistinguishable facial expression. Fig 7 shows the distribution of assessed facial valence in Experiment 2. The horizontal axis shows the facial valence as assessed by participants and the vertical axis shows the normalized number of answers for each posture. The red line histogram shows the result of the intense facial expressions in Experiment 1 as a reference. We calculated the entropy of each distribution, which is shown in the upper left of the histogram. The highest was -43/B and the lowest was 0/A.

**Fig 8:**
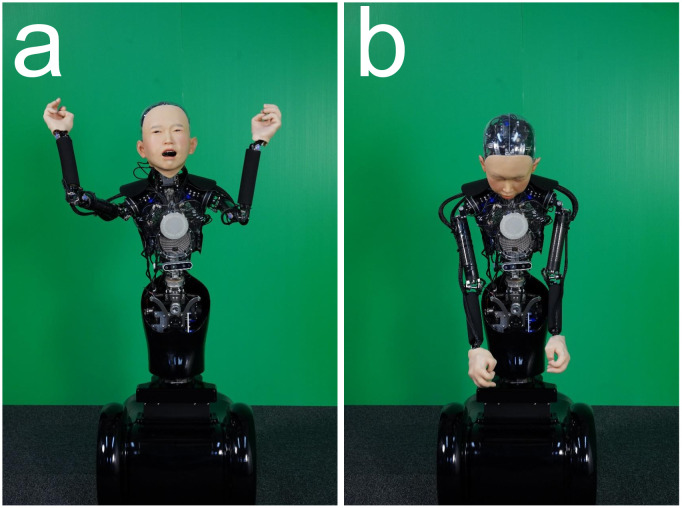
Two representative postures which have distinguishable facial expressions by adding body expressions. (a) The posture -17/A at the highest facial valence / (b) The posture 43/C at the lowest facial valence.

In order to investigate whether the mean values difference of facial valences were affected by HA and AP, we ran a two-way repeated measures ANOVA with HA and AP. For the assessment of facial valence, there were significant main effects of HA (*F* (4, 113) = 23.86, *p* < 0.001, *η*_*p*_^2^ = 0.034) and AP (*F* (2, 113) = 86.16, *p* < 0.001, *η*_*p*_^2^ = 0.202) and there was also a significant interaction effect of the HA/AP (*F* (8, 113) = 9.89, *p* < 0.001, *η*_*p*_^2^ = 0.055). Then, we ran HSD test to verify the difference of the mean values for the 15 postures. Table B in S1 Appendix shows the mean differences for each posture. The highest valence (most positively perceived) posture was HA: -17 degrees and AP: A (Fig 8a, Mean value = 1.89, Standard Error = 0.19), while the lowest valence (most negatively perceived) posture was HA: 43 degrees and AP: C (Fig 8b, M = -1.40, SE = 0.22), with a significant difference of 3.29 between these two postures (*p* < 0.001).

Table 2 shows the percentages of people that assessed each posture as *won* in Experiment 2. AP: A was assessed as *won* between 54.1% and 83.7%, in contrast AP: B and AP: C were assessed as lost (lower than 50%). The result is also consistent with the assessment tendency of the facial valence.

**Table 2:** Percentage of participants that judged each posture as won a game in Experiment 2.

		HA				
		-43	-17	0	17	43
AP	A	61.2	83.7	82.7	54.1	55.1
	B	50.0	36.7	35.7	25.5	5.1
	C	49.0	27.6	42.9	6.1	7.1

Looking at HA: -17, the maximum value (83.7%) was under AP: A, on the contrary, the same HA: -17 was also the minimum value (27.6%) under AP: C. Interestingly, there was a 56.1 points gap between AP: A and AP: C even with the same HA.

Figs A and B in S1 Appendix show the distribution of facial intensity and human-likeness in Experiment 2. The total average of intensity was 6.14 with SE = 0.04 and human-likeness was 6.43 with SE = 0.04. Furthermore, the facial valence had a low correlation with intensity (*r* = 0.163, *p* < 0.001) and with human-likeness (*r* = 0.138, *p* < 0.001).

### Experiment 3

**Fig 9:**
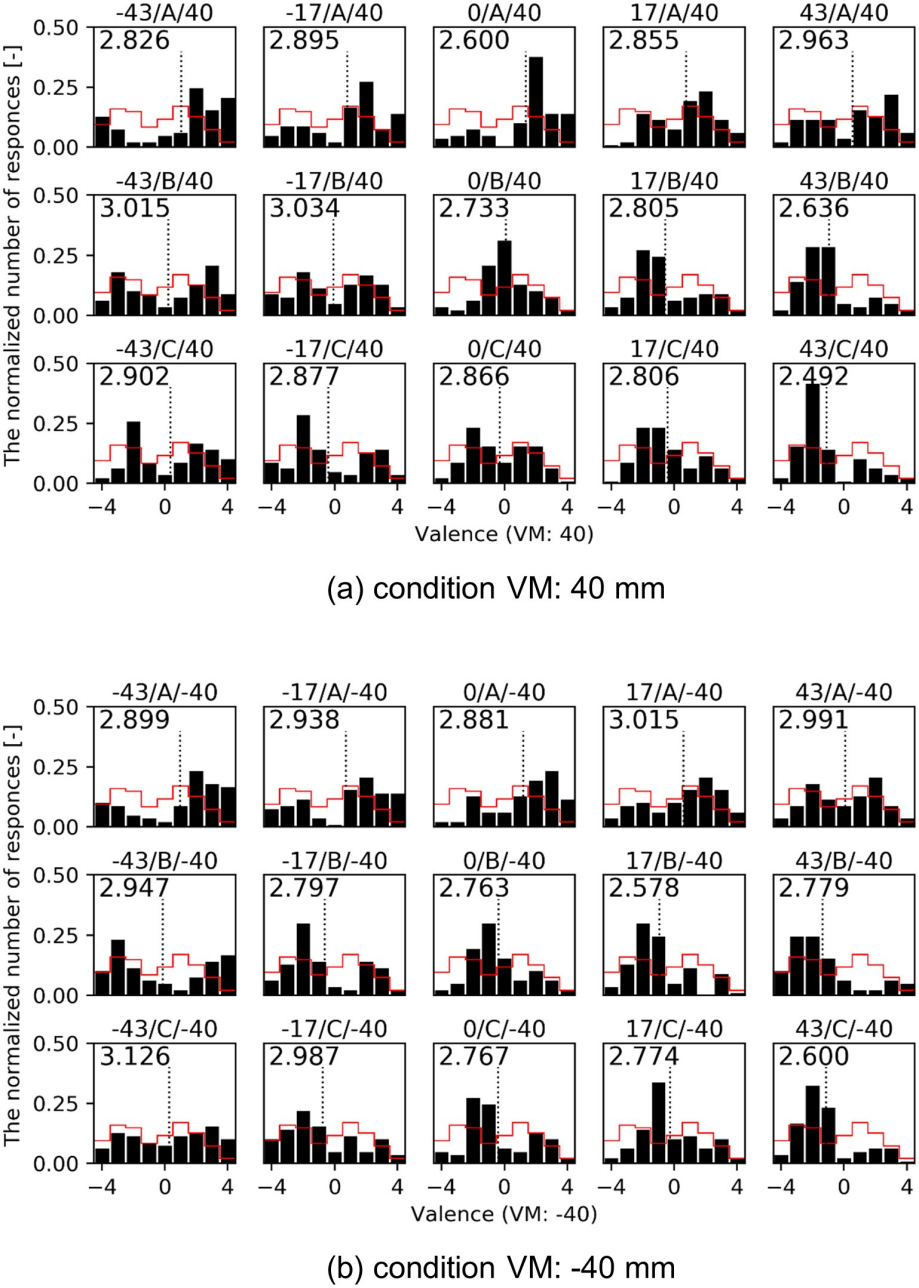
Distributions of the assessed facial valence in Experiment 3. The horizontal axis shows the facial valence as assessed by participants and the vertical axis shows the normalized number of answers at each movement. The dotted line shows the mean facial valence. The red line on the histogram shows the result of intense facial expression in Experiment 1 as a reference. The entropy is shown in the upper left of each histogram.

In Experiment 3, *ibuki*’s movement movies were shown to participants in order to investigate the perception of emotional expressions. We validated that movements contribute to the perception of the indistinguishable facial expression. Fig 9 shows the distribution of assessed facial valence for the condition VM: 40 and -40 mm in Experiment 3. The horizontal axis shows the facial valence as assessed by participants and the vertical axis shows the normalized number of answers for each movement. The red line histogram shows the result of intense facial expression in Experiment 1 as a reference. We calculated the entropy of each distribution, which is shown in the upper left of the histogram. The highest was -43/C/-40 and the lowest was 43/A/40.

In order to investigate whether the mean values difference of facial valence were affected by HA, AP, and VM, we ran a three-way repeated measures ANOVA with HA, AP, and VM. For the assessment of facial valence, there were significant main effects for all three: HA (*F* (4, 113) = 11.68, *p* < 0.001, *η*_*p*_^2^ = 0.020), AP (*F* (2, 113) = 52.82, *p* < 0.001, *η*_*p*_^2^ = 0.044), and VM (*F* (1, 113) = 17.35, *p* < 0.001, *η*_*p*_^2^ = 0.007). Also there were significant interaction effects of the HA/AP (*F* (8, 113) = 3.29, *p* = 0.001, *η*_*p*_^2^ = 0.011) and the AP/VM (*F* (2, 113) = 3.99, *p* = 0.021, *η*_*p*_^2^ = 0.003). On the other hand, no significance was found in the HA/VM (*F* (4, 113) = 0.48, *p* = 0.748, *η*_*p*_^2^ = 0.001) or the HA/AP/VM (*F* (8, 113) = 0.57, *p* = 0.806, *η*_*p*_^2^ = 0.002).

Thus, we ran Tukey’s HSD tests to verify the difference of the mean values at each interaction effect of HA/AP and AP/VM. The result of HA/AP is shown in Table C in S1 Appendix and AP/VM is shown in Table D in S1 Appendix. Under the condition of HA and AP, the highest valence movement was HA: 0 degrees and AP: A (M = 1.28, SE = 0.18), while the lowest valence movement was HA: 43 degrees and AP: B (M = -1.14, SE = 0.17), with a significant difference of 2.42 between these two movements (*p* < 0.001). Also under the condition of AP and VM, the highest valence movement was AP: A and VM: 40 mm (M = 0.90, SE = 0.12), while the lowest valence movement was AP: B and VM: -40 mm (M = -0.69, SE = 0.12), with a significant difference of 1.59 between these two movements (*p* < 0.001).

Table 3 shows the percentages of people that assessed each movement as *won* in Experiment 3.

**Table 3:** Percentage of participants that judged each movement as won a game in Experiment 3.

		HA				
		-43	-17	0	17	43
AP/VM	A/40	69.7	64.5	76.3	63.2	50.0
	B/40	47.4	43.4	46.1	23.7	11.9
	C/40	52.6	28.9	40.8	23.7	18.4
	A/-40	68.4	63.2	75.0	65.8	43.4
	B/-40	39.5	30.3	25.0	14.5	17.1
	C/-40	51.3	31.6	30.3	30.3	13.2

As in Experiment 2, different AP leads to the exact opposite judgement for the game result. For example, under HA: 17, there is a 39.5 points gap between A/40 (63.2%) and B/40 (23.7%) and a 51.3 points gap between A/-40 (65.8%) and B/-40 (14.5%).

Figs C and D in S1 Appendix show the distribution of facial intensity and human-likeness in Experiment 3. The total average of intensity was 6.00 with SE = 0.04 and human-likeness was 6.02 with SE = 0.04. Furthermore, the facial valence had a low correlation with intensity (*r* = 0.164, *p* < 0.001) and with human-likeness (*r* = 0.196, *p* < 0.001).

## Discussion

In the past, research on robots expressing emotions has been based on the assumption that a certain expression is equivalent to a certain emotion (e.g. a smiling indicates happiness). However, there has been no investigation into whether robots can also express ambiguous facial expressions with no clear valence. Additionally, for ambiguous facial expressions, there has been no investigation into whether the addition of expressions such as body expression can make the robot’s emotional valence clearer to humans.

In Experiment 1, we validated that the distributions of valence and intensity were different depending on the facial emotion expressions. In particular, according to the results of the entropy of facial valences, participants could not distinguish the facial valence of the intense emotion as positive or negative. The previous study by Aviezer et al. found that it is difficult to judge the valence of intense facial expressions when only the facial expression can be seen [26]. Our experiment corroborates this phenomenon in the case of androids. The histogram of the facial valence of intense emotion in Fig 5 seems to have two peaks in both sides positive (1) and negative (-3). We believe that this is because the participants were mainly divided into two groups who perceived the intense facial expression positively or negatively. Thus, the entropy of intense got the highest value of the eight emotions.

In Experiment 2 and 3, it was found that both body postures and body movements changed the facial valence of the intense emotion and contributed to the lower entropy in the valence assessments. In other words, postures and movements can improve the perception of the ambiguous facial expression. As mentioned in the result, the maximum difference in the mean facial valences was 3.29 for the posture and 2.42 for the movement. Although the number of facial expressions and postures used in the experiment is different, as a reference, those mean differences are as close as the mean difference of 2.3 points in the previous human study by Aviezer et al., in which they exchanged win and lose body postures against intense face in a tennis match. Looking at the histograms of facial emotional values in Fig 7 of Experiment 2, under postures which had lower entropy than that of the intense facial expression in Experiment 1, such as -17/A (0.486 lower) and 0/A (0.556 lower), those distributions only had a one side peak. On the other hand, under postures which had little lower entropy, such as -43/B and -17/B, those distributions still had two peaks in both positive and negative sides as in Experiment 1. However, -17/B/-40 (the movement of -17/B with VM: -40) tended to be perceived more negatively due to the effect of the downward vertical motion in Experiment 3. Thus, -17/B/-40 had a further lower entropy than that of Experiment 2.

From the *η*_*p*_^2^ values in ANOVA, we can deduce that participants’ assessments were most affected by AP, then HA and, finally, by VM. Our findings are consistent with several previous studies on human emotion expressions. As for AP, when the arms are located vertically upwards or move upwards from the body, the posture tends to be perceived as joy [31, 32] and joy corresponds to positive valence [15]. As for HA, raised head angles are associated with positive emotions such as joy [33]. As for VM, rising upper body motion corresponds to happiness, and sinking body motion corresponds to sadness [34]. However, in this study, we only controlled the displacement and not the speed and acceleration of the movement. It has been found that the human perception of robots is negatively impacted if human-like robots perform robot-like movements which contradict its appearance [35]. This might be the reason why VM comes last. Motion activity, including how fast and smooth a person or android is moving, is said to be one of the important factors for emotion recognition [36].

Since interaction effects were confirmed between AP and HA, and AP and VM from the results of ANOVA, we argue that robot developers should not separately design facial expressions from body expressions when designing robots for the application of human-robot interaction, as there are expressions that cannot be distinguished by the facial expressions alone, such as the intense emotion discussed in this paper. Strong emotions influence human social decision making [37], enhancement on memory [38], and time perception [39]. In the same way, strong emotion expressions by robots are expected to enhance the relationship between humans and robots [40].

One limitation of this study was that *ibuki* was based on a young Asian male child. In past studies on recognition of human emotions, it has been reported that age, gender, and ethnicity can influence emotional perception [41, 42]. Therefore, it is necessary to further investigate how our finding applies to other common humanoids and robots universally. Previous research on humans shows that humans can perceive emotions even from point-light displayed facial expressions or body movements [5, 43]. We expect that this capability is also applicable for the emotion perception of robot faces.

## Conclusion

In this paper, we conducted three experiments by controlling the android face and body components. In Experiment 1, we validated a facial expression with no clear valence. Based on the entropy of the valence distribution, the intense facial expression was the most ambiguous. Then we validated whether the addition of postures or movements could make the android’s facial valence clearer to humans in Experiment 2 and 3. We confirmed the possibility of clearly perceiving the android’s facial expression of intense by adding postures or movements.

## Supporting information

S1 Appendix The summary of the experiment result. (PDF)
