# Supplementary material for: Can an android’s posture and movement discriminate against the ambiguous emotion perceived from its facial expressions?
Source: PLoS One. 2021 Aug 10;16(8):e0254905. doi: 10.1371/journal.pone.0254905 (PMC8354482; doi:10.1371/journal.pone.0254905)
Supplement: S1 Appendix — (PDF) [file pone.0254905.s001.pdf]

Appendix

Entropy calculation

Entropy is a physical property that represents a state of disorder of a system. For this study, the entropy was defined as representing an indistinguishable state of the valence assessment for an expressed facial expression, in other words, the degree of unclarity when participants assessed the facial valence. Here we describe how the entropy was calculated for each facial expression assessment. Taking the example of intense facial expressions, the probability $P(x_i)$ to be assessed as valence x_i was shown in Table A. In this case, the entropy S of Eq. (1) is calculated as follows:

$$
\begin{aligned}
S =& -\sum_{x_i=-4}^{4} P(x_i)\log_2 P(x_i) \qquad (1)\\
=& -0.096\log_2 0.096 - 0.160\log_2 0.160 - 0.149\log_2 0.149\\
& -0.085\log_2 0.085 - 0.117\log_2 0.117 - 0.170\log_2 0.170\\
& -0.128\log_2 0.128 - 0.074\log_2 0.074 - 0.021\log_2 0.021\\
=& \quad 3.032
\end{aligned}
$$

Table A. The probability of participants' answers for each valence of intense

x_i	-4	-3	-2	-1	0	1	2	3	4
$P(x_i)$	0.096	0.160	0.149	0.085	0.117	0.170	0.128	0.074	0.021

Table B. The mean difference of facial valence among 15 postures in Experiment 2.

	-43/A	-43/B	-43/C	-17/A	-17/B	-17/C	0/A	0/B	0/C	17/A	17/B	17/C	43/A	43/B	43/C
-43/A		0.52	0.32	-0.93	0.78	1.21**	-0.80	0.70	0.76	0.21	0.85	1.92***	0.13	2.19***	2.36***
-43/B			-0.20	-1.45***	0.26	0.69	-1.32**	0.18	0.23	-0.31	0.33	1.40***	-0.39	1.67***	1.84***
-43/C				-1.24**	0.46	0.90	-1.11*	0.39	0.44	-0.10	0.53	1.60***	-0.18	1.88***	2.04***
-17/A					1.70***	2.14***	0.13	1.63***	1.68***	1.14*	1.78***	2.85***	1.06*	3.12***	3.29***
-17/B						0.44	-1.57***	-0.07	-0.02	-0.56	0.07	1.14*	-0.64	1.42***	1.58***
-17/C							-2.01***	-0.51	-0.46	-1.00	-0.37	0.70	-1.08*	0.98	1.14*
0/A								1.50***	1.55***	1.01	1.64***	2.71***	0.93	2.99***	3.15***
0/B									0.05	-0.49	0.14	1.21**	-0.57	1.49***	1.65***
0/C										-0.54	0.09	1.16*	-0.62	1.44***	1.60***
17/A											0.63	1.70***	-0.08	1.98***	2.14***
17/B												1.07*	-0.71	1.35**	1.51***
17/C													-1.79***	0.28	0.44
43/A														2.06***	2.22***
43/B															0.16
43/C															

Each label shows HA/AP (e.g. -43/A means HA: -43 and AP: A). For example, the top left mean difference 0.52 is calculated as -43/A - -43/B. *: $p < 0.05$, **: $p < 0.01$, ***: $p < 0.001$.

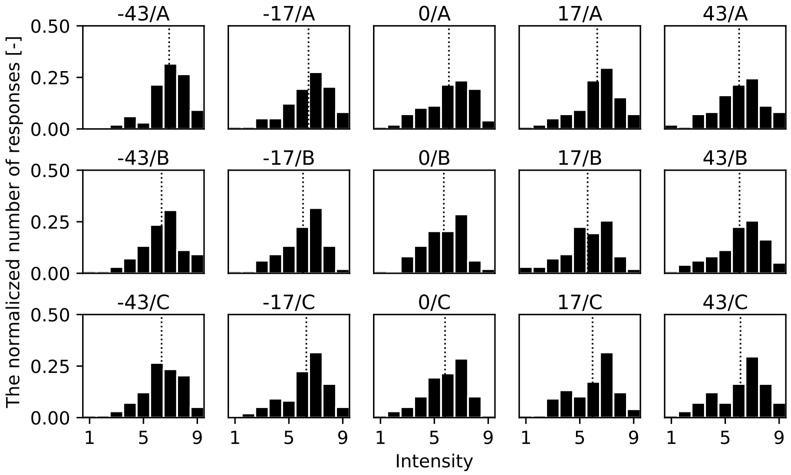

Fig A. Distributions of the assessed facial intensity in Experiment 2. The horizontal axis shows the assessed facial intensity (1 to 9) and the vertical axis shows the normalized number of responses. The dotted line shows the mean facial intensity.

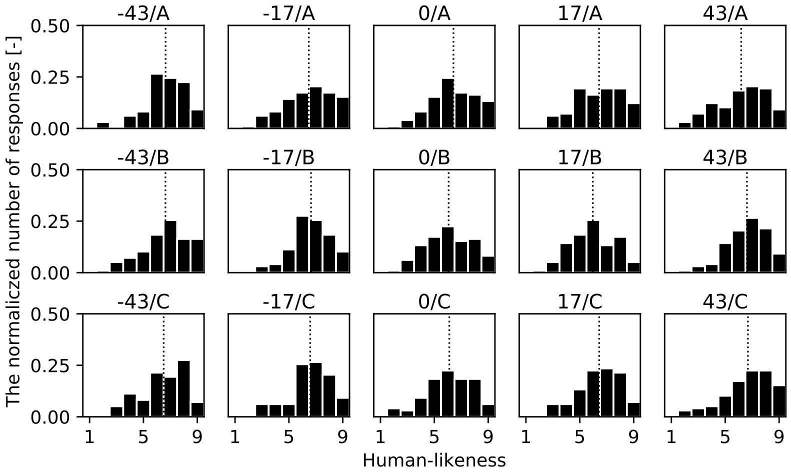

Fig B. Distributions of the assessed facial human-likeness in Experiment 2. The horizontal axis shows the assessed facial human-likeness (1 to 9) and the vertical axis shows the normalized number of responses. The dotted line shows the mean facial human-likeness.

Table C. The mean difference of facial valence among head angles and arm poses in Experiment 3.

	-43/A	-43/B	-43/C	-17/A	-17/B	-17/C	0/A	0/B	0/C	17/A	17/B	17/C	43/A	43/B	43/C
-43/A		0.95*	0.67	0.24	1.35***	1.58***	-0.28	1.14**	1.34***	0.32	1.76***	1.35***	0.70	2.14***	2.12***
-43/B			-0.28	-0.72	0.39	0.62	-1.23***	0.18	0.39	-0.63	0.80	0.39	-0.26	1.19***	1.16***
-43/C				-0.43	0.68	0.91*	-0.95*	0.47	0.67	-0.35	1.09**	0.68	0.03	1.47***	1.45***
-17/A					1.11**	1.34***	-0.51	0.90*	1.11**	0.09	1.52***	1.11**	0.46	1.91***	1.88***
-17/B						0.23	-1.62***	-0.21	-0.01	-1.03**	0.41	-0.00	-0.65	0.80	0.77
-17/C							-1.86***	-0.44	-0.24	-1.26***	0.18	-0.23	-0.88*	0.57	0.54
0/A								1.41***	1.62***	0.60	2.03***	1.62***	0.97*	2.42***	2.39***
0/B									0.20	-0.82	0.62	0.21	-0.44	1.01**	0.98*
0/C										-1.02**	0.41	0.01	-0.64	0.80	0.78
17/A											1.43***	1.03**	0.38	1.82***	1.80***
17/B												-0.41	-1.06**	0.39	0.36
17/C													-0.65	0.80	0.77
43/A														1.45***	1.42***
43/B															-0.03
43/C															

Each label shows HA/AP. *: $p < 0.05$, **: $p < 0.01$, ***: $p < 0.001$.

Table D. The mean difference of facial valence among arm poses and vertical motions in Experiment 3.

	A/40	A/-40	B/40	B/-40	C/40	C/-40
A/40		0.19	1.14***	1.59***	1.27***	1.35***
A/-40			0.96***	1.40***	1.08***	1.16***
B/40				0.44	0.12	0.21
B/-40					-0.32	-0.24
C/40						0.08
C/-40						

Each label shows AP/VM. ***: $p < 0.001$.

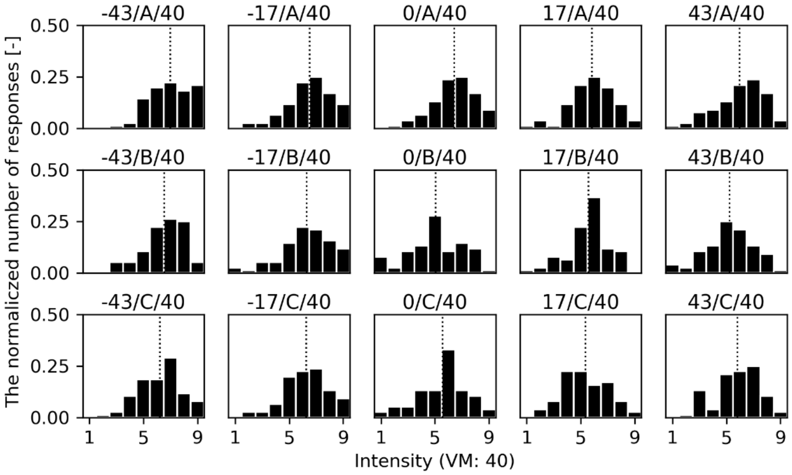

(a) condition VM: 40 mm

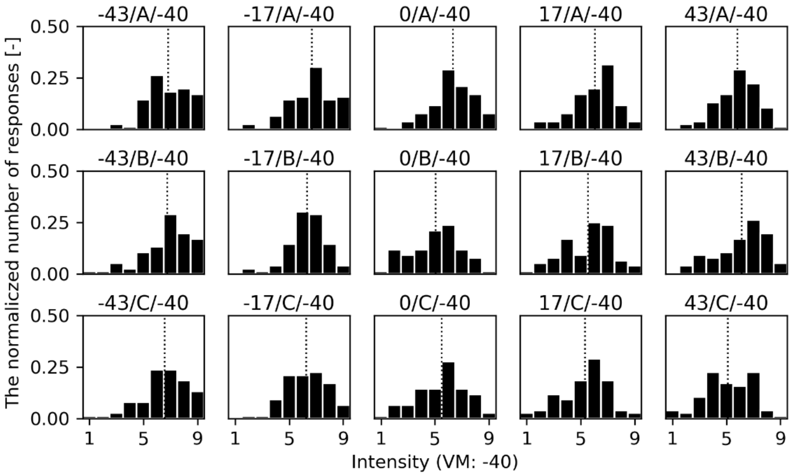

(b) condition VM: -40 mm

Fig C. Distributions of the assessed facial intensity in Experiment 3. The horizontal axis shows the facial intensity as assessed by participants and the vertical axis shows the normalized number of answers at each movement. The dotted line shows the mean facial intensity.

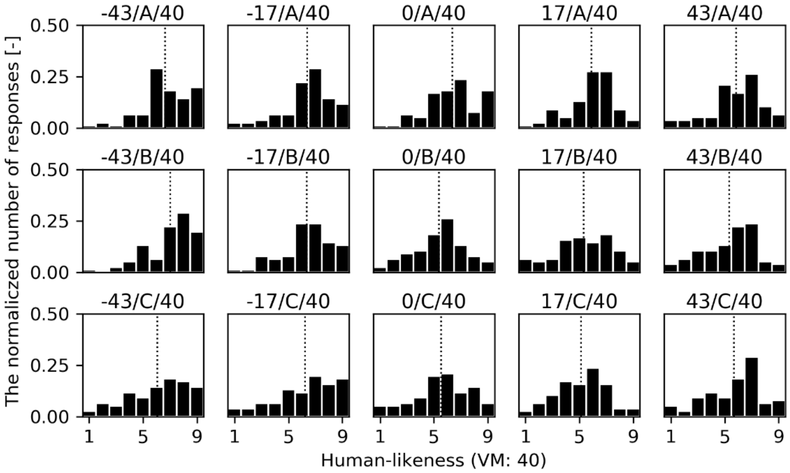

(a) condition VM: 40 mm

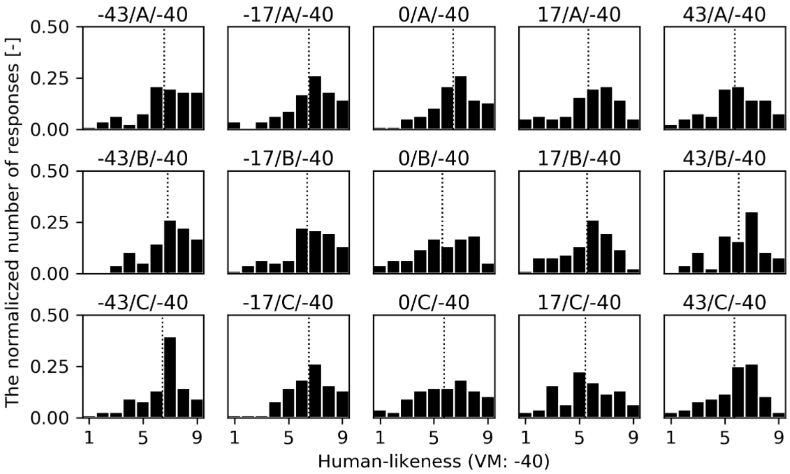

(b) condition VM: -40 mm

Fig D. Distributions of the assessed facial human-likeness in Experiment 3. The horizontal axis shows the facial human-likeness as assessed by participants and the vertical axis shows the normalized number of answers at each movement. The dotted line shows the mean facial human-likeness.
